# Supplementary figures and images for: Language laterality and cognitive skills: does anatomy matter?
Source: Cereb Cortex. 2026 Jun 25;36(6):bhag067. doi: 10.1093/cercor/bhag067 (PMC13298645; doi:10.1093/cercor/bhag067)

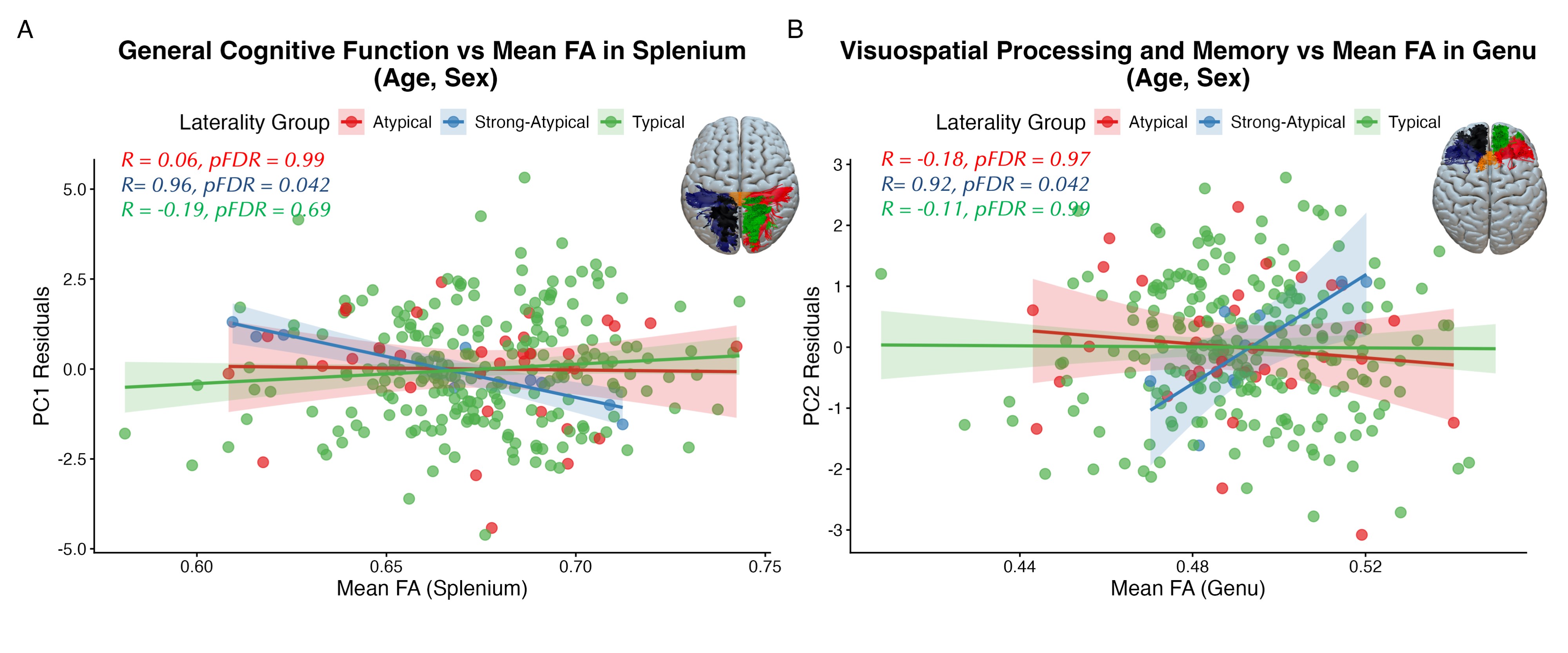

Supplement: Supplementary_Figure_bhag067 [file supplementary_figure_bhag067.jpeg]
